# Entertaining accurate treatment expectations while suffering from chronic pain: an exploration of treatment expectations and the relationship with patient- provider communication

**DOI:** 10.1186/s12913-018-3497-8

**Published:** 2018-09-11

**Authors:** Bianca Wiering, Dolf de Boer, Maarten Krol, Hilda Wieberneit-Tolman, Diana Delnoij

**Affiliations:** 1 Tranzo (Scientific Centre for Transformation in Care and Welfare), Tilburg University, PO Box 90153, 5000 LE Tilburg, The Netherlands; 2 NIVEL (Netherlands institute for health services research), Utrecht, the Netherlands; 3 Pijn Platform Nederland, Leiden, the Netherlands

**Keywords:** Treatment expectations, Treatment goals, Health care provider communication, Shared decision making, Patient reported experience measure

## Abstract

**Background:**

Accurate patient expectations are important to optimise treatment success, especially for complex conditions such as chronic pain. Communication may be the key to managing patient expectations. This study aimed to explore whether health care provider communication influences patient expectations and which communication aspects are most important.

**Methods:**

We conducted secondary analyses on data that had been collected between September and November 2012. 2603 patients suffering from chronic pain were invited to complete a survey.

**Results:**

Although 69.9% of patients achieved or surpassed their treatment goal, 30.2% of patients were unsatisfied. Even though overall health care provider communication and shared decision making were unrelated to patient expectations, several affective communication aspects were related. These aspects were attentive listening, taking enough time, building patient’s trust in the physician’s competence and giving patients the feeling that the physician is doing all he or she can (*p’s* < 0.05).

**Conclusions:**

Even though treatment goals are not always explicitly discussed, patients still form expectations regarding treatment outcomes. Affective communication may be more important for managing patient expectations than sharing information. Building a good therapeutic relationship by showing affective communication may be important to increase the accuracy of patient expectations.

## Background

Patient expectations of treatment outcomes can be very important for achieving optimal treatment success. Patient expectations can not only influence satisfaction after treatment, [1–3] but also patient outcomes [4–6], the number of return visits [7] and self-care [6]. However, research shows that many patients harbour inaccurate expectations regarding treatment outcomes [8–11]. The term inaccurate or unrealistic expectations is used to describe expectations that do not match with what is expected to be achievable with treatment [10, 12, 13]. Patients’ expectations regarding treatment goals can differ widely from those of their physicians [14]. Ensuring that patients entertain accurate expectations of treatment may be especially important for medically complex chronic conditions. Patients suffering from these conditions may need treatment for a long time [15], treatment is often based on self-medication [16, 17] and treatment goals may be less clear. Accurate expectations may prevent these patients from getting discouraged, which may for instance influence medication adherence [6].

A good example of a complex chronic condition is chronic pain. A large survey of 50,000 respondents originating from 15 European countries and Israel showed that as many as one in five adults in Europe may be suffering from chronic pain [15]. Many patients suffer from chronic pain for a number of years [15, 18]. Chronic pain may affect patients’ emotional well-being [15, 19], physical functioning [20], social activities and work [15, 21]. There are many treatment options available, such as anti-inflammatory agents, opioid analgesics, and physical therapy [18, 22, 23]. However, it is not uncommon that treatment is insufficient to treat the pain [15, 24, 25]. For these patients, treatment goals can range from easing or stabilising their pain, to a slower deterioration, or learning to deal with the condition [22, 23]. Even though a cure may not be achievable, a good match between patient expectations and outcomes has been shown to improve patients’ satisfaction [22]. This makes the management of patient expectations a fairly effective way to optimise patients’ satisfaction levels with treatment.

One way to improve the accuracy of patient expectations may be by improving patient-provider communication. Active engagement in shared decision making, patient education or discussing patients expectations could perhaps help physicians clarify what can be expected from treatment and address misconceptions [1, 14]. However, many patients fail to discuss their expectations [26] and identifying patients’ expectations seems to be a challenge for physicians [27, 28]. Furthermore, it is not clear which aspects of communication may be of importance to improve the accuracy of patient expectations and treatment goals.

More knowledge is needed about whether health care provider communication may help to achieve more accurate patient expectations, and if that is the case, which aspects of communication contribute. Although health care providers’ views may also be important for identifying aspects of care which benefit the accuracy of patient expectations, in the present study only the patients’ views were included. By exploring patients’ treatment expectations in relation to patients’ views on communication during consultations, this study aimed to explore the role of health care provider communication and the different aspects of communication in achieving more accurate treatment goals.

Our research questions were:

- Do patients entertain accurate treatment expectations?
- Is better health care provider communication associated with more accurate treatment expectations?
- Which health care provider communication aspects are important in clarifying what patients can expect from treatment?

## Methods

### The umbrella study

This study is based on secondary analysis of data from a bigger study initiated by a Dutch umbrella organisation ‘Pijn Platform Nederland’, with the aim of, among other things, developing a patient reported experience measure [29]. Three of the five current authors were part of the umbrella study. The umbrella study was responsible for recruiting all participants and collecting the data. The following participants and procedure sections are descriptions of how the umbrella study recruited participants and collected data.

### Participants

Four Dutch patient organisations for patients suffering from chronic pain each supplied about 600 randomly chosen member addresses for this study. One patient organisation contacted their 600 randomly chosen members first to gain approval before sharing the addresses. 182 members of this organisation allowed the sharing of their addresses. In the end, 2603 patients suffering from chronic pain were invited to fill in a questionnaire during the period of September–November 2012.

### Procedure

A formal ethical board review was not required for this study, as it did not fall under the Dutch Medical Research Involving Human Subjects Act (WMO) [30]. The National Health Care Institute guidelines were applied during data collection. The guidelines cover privacy and informed consent [31]. Participation was voluntary and anonymous. Patients received an invitation letter either by e-mail or by post from the Dutch umbrella organisation, the website address of the survey, login details and a card that could be sent back if one did not wish to participate. A reminder or thank you note was sent a week after the invitation letter. After 2 weeks, a second reminder was sent, accompanied by a paper version of the survey or thank you note. A fourth and final reminder or a thank you note was sent after 3 weeks.

### Measures

The survey included questions on background characteristics, a patient reported outcome question, and a patient reported experience measure (PREM) [29]. The background characteristics used in this study were age, gender, overall health, and educational attainment.

Several questions and measurement instruments were used to establish how accurate patient expectations were compared to treatment goals (Tables 1 and 2). The first question concerns a patient reported outcome question. The question asked patients whether their level of pain had changed since starting treatment. Answer options ranged from 1 ‘ Yes, the pain has gone’, to 6 ‘Yes, the pain has gotten worse’.

**Table 1:** The first part of the construction of the dependent variable: the merger of the responses to the patient reported outcome question and the treatment goals. (*N* = 585)

	Goal is complete recovery	Goal is decrease in pain	Goal is to stabilise pain
The pain has gone	9^b^	1^c^	0^c^
The pain has (slightly) decreased	36^a^	238^b^	110^c^
The pain is stable	6^a^	83^a^	61^b^
The pain has (slightly) increased	2^a^	23^a^	16^a^

^a^Goal was not achieved

^b^Goal was achieved

^c^Results were better than treatment goal

**Table 2:** The second part of the construction of the dependent variable: the accuracy of patient expectations (too low expectations, accurate expectations and too high expectations).(*N* = 469)

	Goal was not achieved				Goal was achieved				Results were better than goal			
	Pain increased	Pain is stable	Pain decreased	Pain has gone	Pain increased	Pain is stable	Pain decreased	Pain has gone	Pain increased	Pain is stable	Pain decreased	Pain has gone
Patient expected more	29^b^	54^b^	12^b^	0	0	23^c^	52^c^	1^c^	0	0	23^c^	0
Patient expectations matched results	6^a^	11^a^	13^a^	0	0	22^b^	51^b^	1^b^	0	0	24^c^	0
Patient expected less	0	8^a^	8^a^	0	0	4^a^	79^a^	5^a^	0	0	42^b^	1^b^

^a^Too low expectations compared to treatment goals and treatment results

^b^Accurate expectations compared to treatment goals and treatment results

^c^Too high expectations compared to treatment goals and treatment results

Further questions concerning treatment goals and expectations which were part of a PREM [29] were also included. Patients were asked what the goal of their treatment was. Answer options ranged from 1 ‘Complete recovery’, to 4 ‘Learning to cope with the pain’. Patients were also asked whether the treatment results matched their expectations. Answer options ranged from 1 ‘Not at all’, to 4 ‘Yes’. Finally, patients who did not answer ‘Yes’ to the former question were asked why the results did not match their expectations. Answer options were ‘The result was better than I expected’, and ‘The result was not as good as I expected’.

Finally, communication was measured using two subscales from the PREM concerning health care professional communication and shared decision making [29] (Table 3). Patients rated their experiences with health care provider communication from 1 ‘Never’, to 4 ‘Always’.

**Table 3:** Patient and pain characteristics. (*N* = 886)

	N (%)	Mean (SD)
*Age*		
18 to 24 years	8 (.9%)	
25 to 34 years	40 (4.6%)	
35 to 44 years	100 (11.4%)	
45 to 54 years	246 (28.0%)	
55 to 64 years	274 (31.2%)	
65 to 74 years	142 (16.2%)	
75 years or older	68 (7.7%)	
Sex (Female)	633 (72.9%)	
*Educational attainment*		
University (MSc/BSc)	61 (7.2%)	
Higher vocational education (BSc)	238 (28.2%)	
Middle vocational education	188 (22.2%)	
High school/ secondary education	337 (39.9%)	
< High school level	21 (2.5%)	
*Cause of pain*		
Accident	254 (30.9%)	
Disease	168 (20.4%)	
Surgical procedure	78 (9.5%)	
Unknown	178 (21.7%)	
Other	144 (17.5%)	
Duration of pain in years (until 2012)		14.9 (11.9)
*Treatment type*		
Medication	114 (17.7%)	
Injections	47 (7.3%)	
Transcutaneous electrical nerve stimulation	18 (2.8%)	
Surgical procedure	18 (2.8%)	
Physiotherapy	265 (41.2%)	
Cesar*/*Mensendieck practice therapy	17 (2.6%)	
Psychological support	27 (4.2%)	
Other	99 (15.4%)	
*Treatment goal*		
Complete recovery	53 (7.7%)	
Decrease in pain	347 (50.7%)	
Stabilising pain	187 (27.3%)	
Learning to cope with pain	65 (9.5%)	
Other	28 (4.1%)	

### Statistical analyses

Raw data files from the umbrella study were used to conduct the analyses. Participant and pain characteristics, patient expectations and treatment goals were described using univariate analyses. To calculate whether patients’ expectations were accurate compared to the treatment goals in light of the treatment results, the questions regarding whether the results matched patients’ expectations and why the results did not match their expectations were combined. This resulted in a variable ranging from ‘The results were not as good as expected’, to ‘The results were better than expected’. Furthermore, treatment results (i.e. the answers to the patient reported outcome question) and treatment goals were compared to establish whether the treatment goal was achieved. As the patient reported outcome question only gave answer options describing pain progression or relief, the treatment goal answer option ‘Learning to cope with the pain’ was removed from analysis. This resulted in a variable ranging from ‘Goal was not achieved’, to ‘Results were better than treatment goal’ (Table 1). Both these variables were combined to create a variable describing how accurate expectations were (for example: results were better than treatment goal, but patient still expected better. In such a case, the patient entertained too high expectations) (Table 2). The term accuracy is used to describe whether patients expected the result that should have been achieved based on the treatment goals. To explore any associations between communication and treatment expectations, partial Pearson correlations were used^1^. Variables included in the correlation analysis were the variable describing how accurate treatment expectations were (i.e. too low expectations, accurate expectations and too high expectations), and the subscales health care provider communication (an average of 7 items; Cronbach’s alpha = .92) and shared decision making (an average of 4 items; Cronbach’s alpha = .81). The subscales were based on principal component analysis. Principal component analysis was conducted for the umbrella study and was repeated for this study. As factor analysis showed that the Cronbach’s alpha was significantly higher if one item was deleted from the shared decision making scale [29], the item was removed from analysis. To further explore which communication aspects are specifically associated with treatment expectations, all communication items were entered separately in a second partial correlation analysis. Furthermore, all analyses were controlled for sex, age, overall health and educational attainment. Analyses were performed using SPSS 22.0 [32].

## Results

### Response

**Fig. 1:**
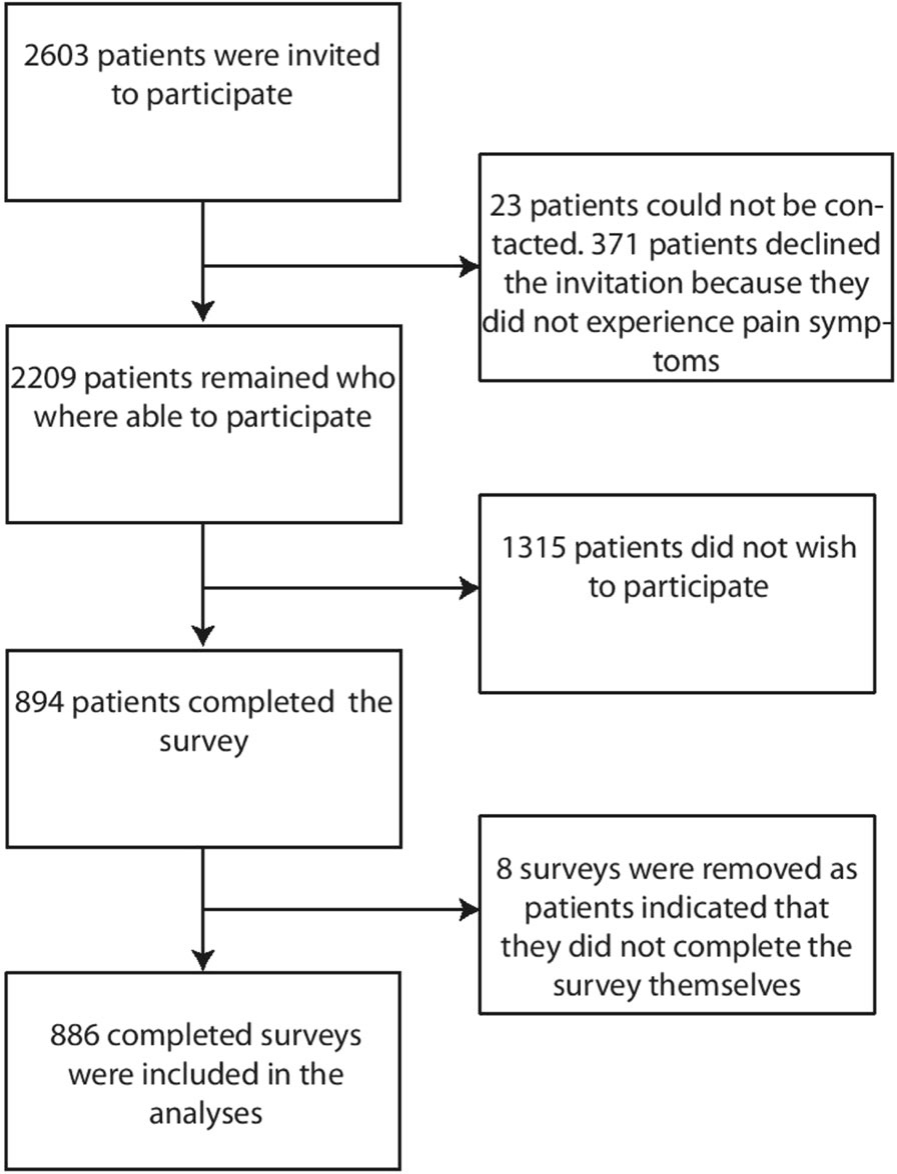
Flow chart

Of the 2603 patients invited to participate, 23 patients could not be contacted because the address was unknown, or the patient had passed away. 371 patients indicated that they would not take part because they did not currently experience pain symptoms. Of the remaining 2209 patients eligible to participate, 894 patients completed the questionnaire. This meant a response rate of 40.5%. Finally, the data of 8 patients were removed because they indicated that they did not complete the questionnaire themselves. A total of 886 patients’ results were included in the analyses (Fig. 1).

### Participants

The age group 55 to 64 years was most prevalent among the participants (Table 3). Most participants were female (72.9%) and completed secondary education (39.9%). Many participants suffered from pain for a long time (Mean: 14.9 years; SD: 11.9 years). Common causes of pain were accidents (30.9%) and disease (20.4%). Additionally, many participants did not know what caused their chronic pain (21.7%). Most participants received physiotherapy (41.2%) or medication (17.7%).

### Patients’ expectations of treatment

Participants indicated that they mostly received treatment with the aim to decrease (50.7%) or stabilise (27.3%) their pain level. For many patients the pain did stabilise (26%), or even decreased to a greater (29.4%) or lesser extend (34%). However, about 41.8% of patients indicated that they had expected better results, while 33.3% of patients achieved a better result than they expected. If the results are compared to the treatment goals as described by the patients, there is still a gap between results and expectations. Although 69.9% of patients achieved or surpassed their treatment goal, and 39.9% of patients did not expect such a great improvement, 30.2% of the patients were still unsatisfied.

### Communication and expectations

Although in only 29.9% of cases treatment goals were discussed in full, just 4 patients indicated that they truly did not know what the goal of their treatment was. Apparently patients do not need much discussion of treatment goals to have an idea about what the goal should be. This is further reflected in the lack of an association between communication and how accurate patients’ expectations are compared to these goals. Both health care provider communication (*r* = −.10, *p* = .08) and shared decision making (*r* = −.06, *p* = .27) were unrelated to the accuracy of patients’ expectations (i.e. whether patients’ expectations match with the treatment goals) (Table 4). However, although overall communication may not be related to patient expectations, certain aspects of communication were related to the level of accuracy of patient expectations. For several communication aspects lower scores were related to too high expectations (i.e. Patients expected better results, even though the treatment goal was achieved or surpassed). Important communication aspects were attentive listening (*r* = −.11, *p* = .05), time available for the patient (*r* = −.14, *p* = .01), whether patients trust their health care provider’s competence (*r* = −.12, *p* = .03), and whether patients felt that their health care provider had done all he or she could (*r* = −.18, *p* = .00).

**Table 4:** The association between communication aspects and the level of patient expectations compared to treatment goals (i.e. Too low, accurate or too high expectations). (*N* = 315)

	Level of patient expectations compared to Treatment goals	
	R	P
Overall health care provider communication	−.10	.08
Shared decision making	−.06	.27
The health care provider listened carefully	−.11	.05
The health care provider spent enough time on the patient	−.14	.01
The health care provider took the patient seriously	−.10	.07
The patient trusts the health care providers’ competence	−.12	.03
The health care provider paid attention to emotional problems	.05	.39
The health care providers explained everything clearly	−.08	.15
The health care provider has done all he or she could	−.18	.00
The health care provider discussed what can be expected of treatment	−.02	.78
The health care provider provided information on treatment options	−.07	.20
The patient took part in the decision making process	−.06	.31
The health care provider took into account the patient’s preferences while deciding for a treatment	−.05	.34

Analyses were controlled for sex, age, overall health and educational attainment

## Discussion and conclusion

### Discussion

For conditions such as chronic pain, treatments are often not a cure and treatment goals are not always clear cut. However, accurate expectations of treatment may increase patients’ satisfaction [22]. This study therefore first aimed to explore whether patients have a clear idea of what should be achieved with treatment. The present study found that although treatment goals were seldom fully discussed, almost all patients had ideas about what the goal of their treatment was. However, even if this treatment goal was achieved or surpassed, many patients still entertained higher or lower expectations than the achieved result. Perhaps imagining what it would feel like to achieve a treatment goal may still be fairly difficult for patients. Inaccurate expectations are fairly common among patients, for example among patients undergoing surgery [10, 11]. However, surgery usually consists of one operation with the aim of improving health aspects such as functioning [33], pain level [11] or weight [10, 34]. Treatments of chronic conditions such as chronic pain are not always given with the aim to improve and may even only focus on coping [22, 23]. As possible outcomes are not limited to improvement and treatment outcomes may be uncertain, it is perhaps even more difficult to entertain accurate expectations.

As apparently not all patients seem to know what to expect from treatment, the present study tried to explore whether better health care provider communication may help achieve more accurate treatment expectations. This study found that although improving health care provider communication overall may not benefit the accuracy of patient expectations, several aspects of communications were found to be related to patient expectations. Perhaps the lack of influence from some communication aspects counterbalanced the few communication aspects which did have an effect on patients’ expectations. More remarkable is the difference between the communication aspects which did not influence expectations and the communication aspects which did. Many of the communication aspects which were not significantly related to expectations concerned instrumental communication aspects. Instrumental communication focuses on the patient’s cognitive need to be informed [35]. Important aspects of instrumental communication are gathering data by asking questions and providing information [36]. Due to its informative nature, one would expect that showing more instrumental communication would increase the accuracy of patient expectations. This, however, appears not to be the case. Instead, affective communication may be the key to accurate expectations. Affective communication focuses on building a therapeutic relationship between the health care provider and the patient [36]. In this case, the aspects of affective communication that seemed to matter centred around attentive listening, taking enough time, building patient’s trust in the physician’s competence, and giving patients the feeling that the physician is doing all he or she can. These communication aspects are often seen as important factors that contribute to the building of the patient’s trust in a health care provider [37, 38]. It is perhaps not very surprising that patients are more willing to adhere to treatment recommendations if they trust their health care provider [37]. Perhaps this is also the case for treatment expectations. Possibly the affective communication contributes to the building of a level of trust which ensures that patients more easily trust what their health care provider tells them.

However, there appears to be a fine line between good communication, too much communication and too little. Low scores on the important communication aspects were related to expectations that were too high, while high scores were related to too low expectations. Apparently the perfect level of communication is somewhere in the middle. The relationship between low patient expectations and good affective communication may be a sign that it worries patients if health care providers show too much empathy and therefore come across as concerned. Alternatively, research shows that patients usually entertain too high expectations to begin with [10, 11]. As patients are more likely to recall advice correctly if it has been discussed for longer [39], taking more time may have ensured that patients better recall their physician’s advice and lower their expectations accordingly. The relationship between too high expectations and little affective communication may be due to cognitive dissonance, where individuals change or distort two dissonant ideas to make them more consonant [40]. In this case, patients’ disappointment with the treatment results may have coloured their recollection of their health care provider’s communication.

### Limitations

There are several limitations that need to be taken into account. First, although the health care providers’ views on treatment goals and communication may be equally important to identify the accuracy of patient expectations and important communication aspects, this study reports secondary analyses of data that only contained questionnaires completed by patients.

Second, the data used for the secondary analyses was collected in 2012. Although some of the data may be slightly outdated, research suggests that health care provider communication and especially psychosocial communication, have not improved much over time [41]. Furthermore, treatment for chronic pain is still considered ineffective for many patients [42]. As a cure is still unlikely for many patients, ensuring that patients entertain accurate expectations should still be a priority. It is therefore unlikely that the use of slightly older data has impacted the relevance of the study’s results.

Third, research shows that patients have problems recalling what has been discussed after consultations [9, 39]. These recall problems may also be applicable to recalling communication and treatment goals. Perhaps a more objective method may be to record the consultations [43].

Fourth, the response rate was fairly low and no data is available of the patients who did not respond. It is therefore not possible to compare the participant group to the non-response group to check the representativeness of the participant group.

Additionally, all participants were affiliated with organisations concerned with the condition of patients suffering from chronic pain. This also may have affected the representativeness of the participant group, as they were arguably more informed on the subject of pain treatment than random patients suffering from chronic pain.

### Practice implications

The results of this study have several implications for chronic pain consultations and research. First, this study showed that patients feel that treatment goals are not always discussed during consultations. As patients still develop ideas about what should be achieved by undergoing a certain treatment, actively discussing treatment goals may be a useful start in clarifying what patients can expect.

Furthermore, even if patients achieved the treatment goals they indicated on the questionnaire, they still not always felt that their expectations were met. Perhaps they still had personal goals which were not achieved even after achieving their treatment goals, or possibly they are not sure how achieving the treatment goal should feel. Either way, it may be helpful to set and clarify treatment goals by using aids such as patient reported outcome measures. Scores on health dimensions may help patients to visualise and track their progress [44, 45].

Alternatively, physicians could personalise treatment goals by discussing with patients which activities that are influenced by pain the patient would like to be able to do again. For example, going out to dinner with friends after a work day, or going to the zoo with their children. The success of the treatment is measured by establishing to what extent the patient is able to do the activities. The activity should of course be achievable. This would not only help patients get a better grip on what could and should be achieved, make treatment goals more relevant to patients and raise awareness of when a goal has been achieved, it could also help physicians personalise treatment as part of a more person-centred approach [46]. Such a personal approach may help to further build on the therapeutic relationship. As our results show that communication focused on relationship building influences patients’ expectations, personalising treatment may improve the accuracy of expectations not only via further clarification of treatment goals, but also via an increasingly good relationship.

Furthermore, the results show that there are some communication aspects which may influence how accurate patient expectations are. Apparently, affective communication aspects such as attentive listening, taking enough time, building patient’s trust in the physician’s competence, and giving patients the feeling that the physician is doing all he or she can may influence what patients expect from treatment. However, although studies concerning patient-physician communication call for an increase in behaviours such as taking enough time [47, 48], building trust [49] and listening [50], this study indicates that not only too little of these behaviours may have consequences, but also too much. Further research may be needed to investigate how an excess of positive communicative behaviours influences patient perspectives and outcomes.

Finally, theoretically [51] and empirically [52, 53], there is an association between patient expectations and patient satisfaction. In our study we have not measured satisfaction. We only know whether the patients considered their results better, similar or worse than they expected it to be. It is conceivable that chronic patients who consider their results better than expected, are also more satisfied. Future studies in this patient population could shed more light on this.

### Conclusion

The goals of treatments meant to treat chronic pain are often not fully discussed with patients during consultations. Even though treatment goals may not be explicitly discussed by their physician, patients still form expectations regarding treatment outcomes. These expectations do not tend to be very accurate. Patients can entertain both too high and too low expectations. Even if their treatment goal has been achieved, patient expectations are not always fulfilled. Although overall health care provider communication and shared decision making do not appear to affect the accuracy of patients’ expectations, several affective communication aspects may help patients achieve more accurate expectations. These communication aspects were attentive listening, taking enough time, building patient’s trust in the physician’s competence, and giving patients the feeling that the physician is doing all he or she can. Demonstrating the right level of these behaviours may be a balancing act, as both too much and too little may affect patient expectations for the worse. Further research is needed to investigate the effect of demonstrating too much of the above mentioned communication aspects. Furthermore, personalising treatment goals may help patients understand the treatment goals better and raise awareness of when treatment goals are achieved.

## Abbreviations

PREM: Patient reported experience measure
WMO: Dutch Medical Research Involving Human Subjects Act

## Footnotes

1 Due to a low number of complete cases, partial correlations are reported in the present paper. However, similar results were obtained using ordinal logistic regression analysis.
